# Post-stroke enriched auditory environment induces structural connectome plasticity: secondary analysis from a randomized controlled trial

**DOI:** 10.1007/s11682-022-00661-6

**Published:** 2022-03-29

**Authors:** Aleksi J. Sihvonen, Seppo Soinila, Teppo Särkämö

**Affiliations:** 1 Cognitive Brain Research Unit, Department of Psychology and Logopedics, Faculty of Medicine, University of Helsinki, Turku, Finland; 2 School of Health and Rehabilitation Sciences, Queensland Aphasia Research Centre and UQ Centre for Clinical Research, The University of Queensland, Brisbane, Australia; 3 Neurocenter, Turku University Hospital and Division of Clinical Neurosciences, University of Turku, Turku, Finland

**Keywords:** Music, Environmental enrichment, Stroke, Rehabilitation, Structural connectivity

## Abstract

Post-stroke neuroplasticity and cognitive recovery can be enhanced by multimodal stimulation via environmental enrichment. In this vein, recent studies have shown that enriched sound environment (i.e., listening to music) during the subacute post-stroke stage improves cognitive outcomes compared to standard care. The beneficial effects of post-stroke music listening are further pronounced when listening to music containing singing, which enhances language recovery coupled with structural and functional connectivity changes within the language network. However, outside the language network, virtually nothing is known about the effects of enriched sound environment on the structural connectome of the recovering post-stroke brain. Here, we report secondary outcomes from a single-blind randomized controlled trial (NCT01749709) in patients with ischaemic or haemorrhagic stroke (N = 38) who were randomly assigned to listen to vocal music, instrumental music, or audiobooks during the first 3 post-stroke months. Utilizing the longitudinal diffusion-weighted MRI data of the trial, the present study aimed to determine whether the music listening interventions induce changes on structural white matter connectome compared to the control audiobook intervention. Both vocal and instrumental music groups increased quantitative anisotropy longitudinally in multiple left dorsal and ventral tracts as well as in the corpus callosum, and also in the right hemisphere compared to the audiobook group. Audiobook group did not show increased structural connectivity changes compared to both vocal and instrumental music groups. This study shows that listening to music, either vocal or instrumental promotes wide-spread structural connectivity changes in the post-stroke brain, providing a fertile ground for functional restoration.

## Introduction

Recovery after stroke is based on different neural levels ranging from single neurons to wide-spread functional networks (Cramer, 2008). The hypoperfusion depriving the brain of oxygen and nutrients leads not only to a cascade of cellular and biochemical processes resulting in permanent neural damage, but also in growth of neurites and formation of new synapses to rebuild and remodel the injured networks, contributing to functional restoration (Carmichael, 2006; Cramer, 2018). Since neurogenesis has no known clinically meaningful role in adult brain recovery, the post-stroke neuroplasticity changes lay the foundation for recovery (Cramer, 2008; Cramer et al., 2011; Nudo, 2013).

The neuroplasticity changes supporting recovery of function can be enhanced by increasing stimulation from the environment (Baroncelli et al., 2010), termed environmental enrichment (EE). In general, EE involves organization of the rehabilitation environment and provision of equipment to facilitate voluntary engagement in physical, cognitive, and social activities that provide complex stimulation (Nithianantharajah & Hannan, 2006; Rosenzweig et al., 1978). In animal research, this usually involves housing multiple animals together in a large cage equipped with different toys and enhanced novelty and complexity compared to standard conditions (Zhang et al., 2017).

Evidence from animal studies has shown that EE improves post-stroke cognitive and motor recovery (Nithianantharajah & Hannan, 2006), and that multimodal (e.g., auditory-visual) stimulation is superior to unimodal stimulation (Maegele et al., 2005). Despite this evidence, only few studies have assessed the effects of EE in human stroke patients to date. Studies utilizing a communal and individual EE providing, for example, games, reading material, music and audiobooks, have reported increased levels of physical, cognitive or social activity (Janssen et al., 2014; Rosbergen et al., 2017, 2019). In patients with various neurological disorders, similar communal and individual EE has been shown to improve mood and functional / cognitive abilities (Khan et al., 2016). However, the largest to date (N = 193) trial of a patient-driven EE model did not find significant clinical improvements in these domains in stroke patients (Janssen et al., 2021). The experiences from these studies highlight the importance of EE elements, which are engaging, personally tailored and easily accessible, and which can be provided in sufficient quantity that can result in suboptimal rehabilitation intensity to bring about a behavioural change (Krakauer et al., 2012; Murphy & Corbett, 2009).

Rehabilitation environment can also be enriched by using selected components of EE (Percaccio et al., 2007). One of the potential components of EE is music, which has the capacity to enhance mood and arousal, facilitate verbal and non-verbal (emotional) communication and social interaction, engage multiple cognitive and motor functions, and provide reward and motivation to learn and train (Särkämö & Sihvonen, 2018). Advanced neuroimaging studies on healthy participants have provided evidence that music listening modulates a wide-spread network in the brain, comprising bilateral temporal, frontal, parietal, and subcortical regions in healthy subjects (Alluri et al., 2012; Brattico et al., 2011; Koelsch, 2010, 2014; Schmithorst, 2005; Toiviainen et al., 2014; Zatorre & Salimpoor, 2013). In early subacute stroke patients, music listening activates a similar network of brain regions (Sihvonen et al., 2017b). These observations have provided the initial impetus for studying music in the context of neurological rehabilitation (Sihvonen et al., 2017a) as a form of easily applicable auditory EE that can increase activity-dependent neuroplasticity providing a fertile ground for recovery (Murphy & Corbett, 2009; Särkämö & Soto, 2012).

In stroke patients, daily music listening during the subacute post-stroke stage has been shown to improve cognitive and emotional recovery (Baylan et al., 2020; Särkämö et al., 2008) and to induce structural neuroplasticity changes in frontolimbic regions (Särkämö et al., 2014) compared to standard care and to daily audiobook listening as a control intervention. Recently, using data from the current randomized controlled trial (RCT) pooled together with data from our previous trial (Särkämö et al., 2008), we compared the effects of daily listening to vocal music, instrumental music, and audiobooks and found that the vocal (sung) component seems to be crucial for the rehabilitative efficacy of music. As the primary neuropsychological outcome, vocal music listening improved post-stroke verbal memory recovery compared to instrumental music and audiobooks (Sihvonen et al., 2020). Moreover, as secondary outcomes, vocal music listening improved the recovery of post-stroke language skills (Sihvonen et al., 2020), increased grey matter volume in left temporal regions, strengthened resting-state functional connectivity of the left temporoparietal parts of the language and default mode networks, and enhanced fractional anisotropy (FA) of the left frontal aslant tract (FAT) and stimulus-specific activation of its superior frontal termination areas (Sihvonen et al., 2020; Sihvonen, Pitkäniemi et al., 2021; Sihvonen, Ripollés et al., 2021) compared to audiobooks, suggesting that the behavioural benefits of vocal music listening are coupled with structural and functional reorganization of the left hemisphere language network.

Numerous animal studies have shown that EE ameliorates the consequences of brain injury by promoting structural white matter recovery, resulting in modulation of neural circuits and improved neurological function (Forbes et al., 2020; Gibson et al., 2014; Purger et al., 2016). Despite this evidence, studies on patients on the effects of any form of EE on the structural connectivity after brain injury (e.g., stroke) are still largely lacking. This information would greatly improve our understanding of the prerequisites of effective EE in treating patients, providing, for example, crucial information on its neural mechanisms. While we have previously shown that post-stroke vocal music listening enhances microstructural properties (FA) of the left FAT (Sihvonen, Ripollés et al., 2021), a white matter pathway integral to the language network, the broader structural connectivity changes potentially induced by music as a form of auditory EE in other white matter tracts beyond the language network remain unexplored.

Here, we set out to determine the whole-brain structural connectome changes across both hemispheres induced by post-stroke vocal music listening as a secondary analysis from the diffusion-weighted imaging (DWI) MRI data from our RCT described above (Sihvonen et al., 2020; Sihvonen, Pitkäniemi et al., 2021; Sihvonen, Ripollés et al., 2021), using a sample of 38 acute stroke patients with a 3-month follow-up. More specifically, we carried out connectometry analysis utilizing quantitative anisotropy (QA), which has shown greater sensitivity than conventional single-tensor based or tract-based analysis (Yeh, Badre et al., 2016). Connectometry uses permutation testing to identify group differences in white matter tracts, and has recently been used to uncover white matter connectometry of word production (Hula et al., 2020) and verb retrieval (Dresang et al., 2021) in post-stroke aphasia. Based on the previous functional MRI results from the baseline (pre-intervention) stage of our trial, which showed that music listening activates bilateral frontotemporal, parietal, and subcortical regions in early subacute stage stroke patients (Sihvonen et al., 2017b), we hypothesized that vocal music listening would induce longitudinal (baseline to 3-month stage) structural connectivity changes bilaterally in frontotemporal and parietal regions. Furthermore, we hypothesized that also instrumental music would induce similar structural connectivity changes, although less than vocal music in the left-lateralized language-related tracts.

## Methods

### Subjects and study design

Fifty stroke patients were recruited from the Turku University Hospital between 2013 and 2016 for a three-arm RCT (Clinicaltrials.gov: NCT01749709). Inclusion criteria were acute unilateral ischaemic or haemorrhagic stroke; right-handedness; < 80 years of age; capability to communicate in Finnish and ability to co-operate; residence in Southwest Finland; and normal hearing. Exclusion criteria were prior neurological or psychiatric disease, and substance abuse. The study was approved by the Ethics Committee of the Hospital District of Southwest Finland and performed in conformance with the Declaration of Helsinki. All patients gave an informed consent and received standard stroke treatment and rehabilitation. Baseline MRI imaging and behavioural assessments were performed < 3 weeks post-stroke (mean 12 days, SD 5.5). Patients were then randomized to vocal music (VMG, N = 17), instrumental music (IMG, N = 17), and audiobook groups (ABG, N = 16). The randomization was stratified for lesion laterality (left/right) and performed as block randomization (10 blocks of three consecutive patients for left and right lesions), with the order within the blocks being drawn by a random number generator. The randomization list was generated by a laboratory engineer not involved in the data collection and the persons performing the patient recruitment had no access to it.

During the study, six patients were excluded due to refusal to participate at follow-up and six patients due to incomplete MRI data. The remaining thirty-eight patients (15 female and 23 male, mean age 56.1 years SD 13.4) completed the 3-month MRI and behavioural assessments and were included in statistical analyses (VMG, N = 12; IMG, N = 15; ABG, N = 11; Table 1). The groups were relatively well balanced between stroke-relevant clinical variables such as stroke type (infarct/haemorrhage) (P = 0.398), lesion laterality (P = 0.676), lesion volume (P = 0.712) and stroke severity according to the National Institutes of Health Stroke Scale (NIHSS) scores at the acute stage (< 7 days), F(22,38) = 0.872, P = 0.627; Wilk's Λ = 0.442 (individual categories P = 0.153–0.994; see Table 1). None of the patients received endovascular stroke treatments and only one patient received thrombolytic therapy.

**Table 1:** Baseline demographic and clinical characteristics of the patients

	**Vocal music group (N = 12)**	**Instrumental music group (N = 15)**	**Audiobook group (N = 11)**	***p* value**
Demographic				
Sex (male/female)	5/7	11/4	7/4	0.239 (χ^2^)
Age (years)	58.5 (30.0)	55.0 (12.0)	61.0 (20.3)	0.218 (*F*)
Education (years)	14.8 (3.6)	13.0 (6.0)	11.5 (5.3)	0.450 (*F*)
Music background (pre-stroke)				
Formal music training^a^	0.0 (0.0)	0.0 (0.0)	0.0 (1.0)	0.218 (H)
Instrument playing^a^	0.0 (5.0)	0.0 (3.0)	0.0 (5.0)	0.762 (H)
Music listening prior to stroke^a^	5.0 (0.8)	5.0 (0.0)	5.0 (3.0)	0.265 (H)
Clinical				
Stroke type (infarct/haemorrhage)	10/2	9/6	7/4	0.398 (χ^2^)
Verbal fluency^b^	7.5 (8.8)	9.0 (9.5)	10.0 (5.0)	0.715 (H)
Naming^c^	18.5 (3.3)	18.0 (3.0)	18.0 (4.0)	0.444 (H)
Auditory Comprehension^d^	30.0 (2.5)	29.0 (6.0)	27.0 (10.0)	0.112 (H)
Amusia Overall^e^ (no/yes)	5/7	9/6	2/9	0.103 (χ^2^)
Lesion laterality (left/right)	6/6	7/8	7/4	0.676 (χ^2^)
Lesion volume in cm^3^	29.0 (71.1)	45.3 (88.3)	18.5 (11.2)	0.712 (*F*)

Data are median (IQR) unless otherwise stated. Significant group differences are shown in bold

*F* one-way ANOVA, *H* Kruskal–Wallis test, *χ*^2^ chi-square test

^a^Likert scale 0–5 (0 = never, 1 = rarely, 2 = once a month, 3 = once a week, 4 = 2–3 times a week, 5 = daily)

^b^Classification based on Verbal Fluency Test

^c^Classification based on shortened Boston Naming test

^d^Classification based on shortened Token Test

^e^Classification based on the MBEA Scale & Rhythm subtest average score (< 75% cut-off)

### Intervention

The patients were individually contacted by a music therapist who informed them of their group allocation after baseline assessments. Other researchers were blinded to the group allocation of the patients. The music therapist provided the patients with a portable MP3 player, headphones, and a collection of listening material individually selected to match the music or literature preferences of the patient as closely as possible. The listening material was vocal music with sung lyrics in VMG, instrumental music (with no sung lyrics) in IMG, and narrated audiobooks (with no music) in ABG. All material was in a language that the patients understood best. The patients were instructed to listen to the allocated material by themselves daily (min. 1 h per day) in the hospital or at home from 3 weeks post-stroke (i.e., after the baseline assessments) until the 3-month follow-up assessments, and they were asked to keep a listening diary. During the intervention period, the music therapist kept regular contact with the patients to encourage listening, provide more material, and help with the equipment if needed. The intervention has been described in more detail elsewhere (Sihvonen et al., 2020; Sihvonen, Ripollés et al., 2021).

### MRI data acquisition and reconstruction

Patients were scanned on a 3 T Siemens Magnetom Verio scanner (Siemens Healthcare, Erlangen, Germany) with a standard 12-channel head matrix coil at the Department of Radiology, Turku University Hospital. The MRI protocol comprised high-resolution T1-weighted anatomical images and DWI (TR = 11700 ms, TE = 88 ms, acquisition matrix = 112 × 112, 66 axial slices, voxel size = 2.0 × 2.0 × 2.0mm^3^) with one non-diffusion weighted volume and 64 diffusion weighted volumes (b = 1000 s/mm^2^).

The DWI data were reconstructed in the Montreal Neurological Institute (MNI) space using q-space diffeomorphic reconstruction (QSDR) (Yeh & Tseng, 2011) that allows the construction of spin distribution functions (SDFs) (Yeh et al., 2010). The b-table was checked by an automatic quality control routine to ensure its accuracy (Schilling et al., 2019). Normalization was carried out using the anisotropy map of each participant and a diffusion sampling length ratio of 1.25 was used. The data output was resampled to 2 mm isotropic resolution. Quality of the normalization was inspected using the R^2^ values denoting goodness-of-fit between the participant’s anisotropy map and template as well as inspecting the anatomical localisation of each participant’s forceps major and minor to confirm the normalization quality (Hula et al., 2020). The restricted diffusion was quantified using restricted diffusion imaging (Yeh et al., 2017) and QA was extracted as the local connectome fingerprint (Yeh, Vettel et al., 2016) and used in the connectometry analysis.

### Data availability

Anonymized data reported in this manuscript are available from the corresponding author upon reasonable request and subject to approval by the appropriate regulatory committees and officials.

### Statistical analysis

Diffusion MRI connectometry (Yeh, Badre et al., 2016) analyses were carried out using DSI Studio (http://dsi-studio.labsolver.org, version April 7 2021). Three multiple regression models were used to identify positive local connectome changes across time (3 months > Acute) between the VMG, ABG and IMG. Local connectomes with T-score exceeding 3 were selected and tracked using a deterministic fiber tracking algorithm (Yeh et al., 2013) to obtain correlational tractography. The tracks were filtered by topology-informed pruning (Yeh et al., 2019) with 4 iterations, and a length threshold of 20 voxel distance was used to identify significant tracts. Bootstrap resampling with 2000 randomized permutations was used to obtain the null distribution of the track length and estimate the false discovery rates (FDR).

## Results

**Fig. 1:**
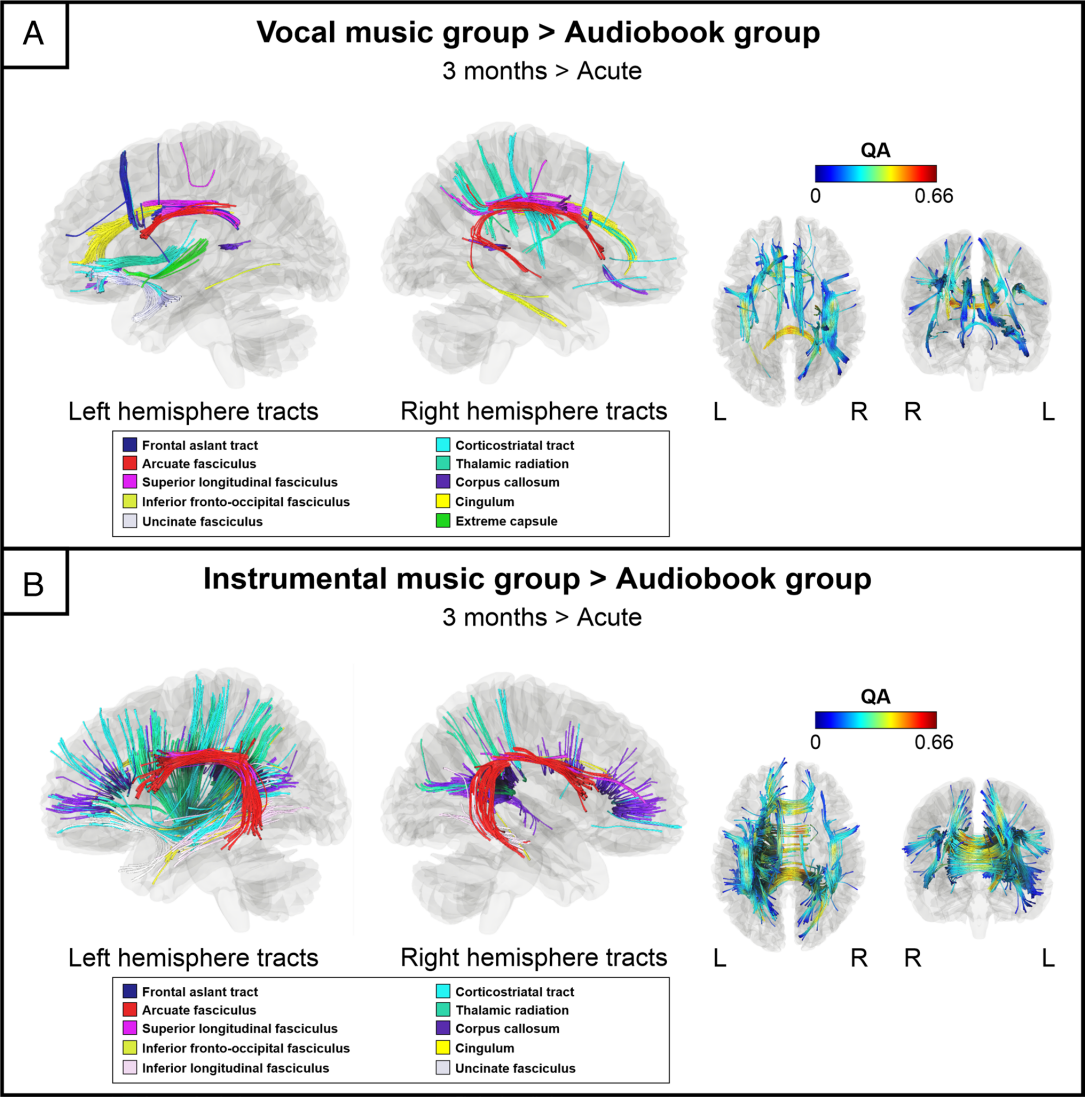
Structural white matter connectometry changes (3 months > Acute). Significant changes in connectometry showing increased structural white matter connectivity between (**A**) VMG and ABG (3 months > Acute) and (**B**) IMG and ABG (3 months > Acute). ABG = Audiobook group, IMG = Instrumental music group, L = left, QA = Quantitative anisotropy, R = right, VMG = Vocal music group

**Fig. 2:**
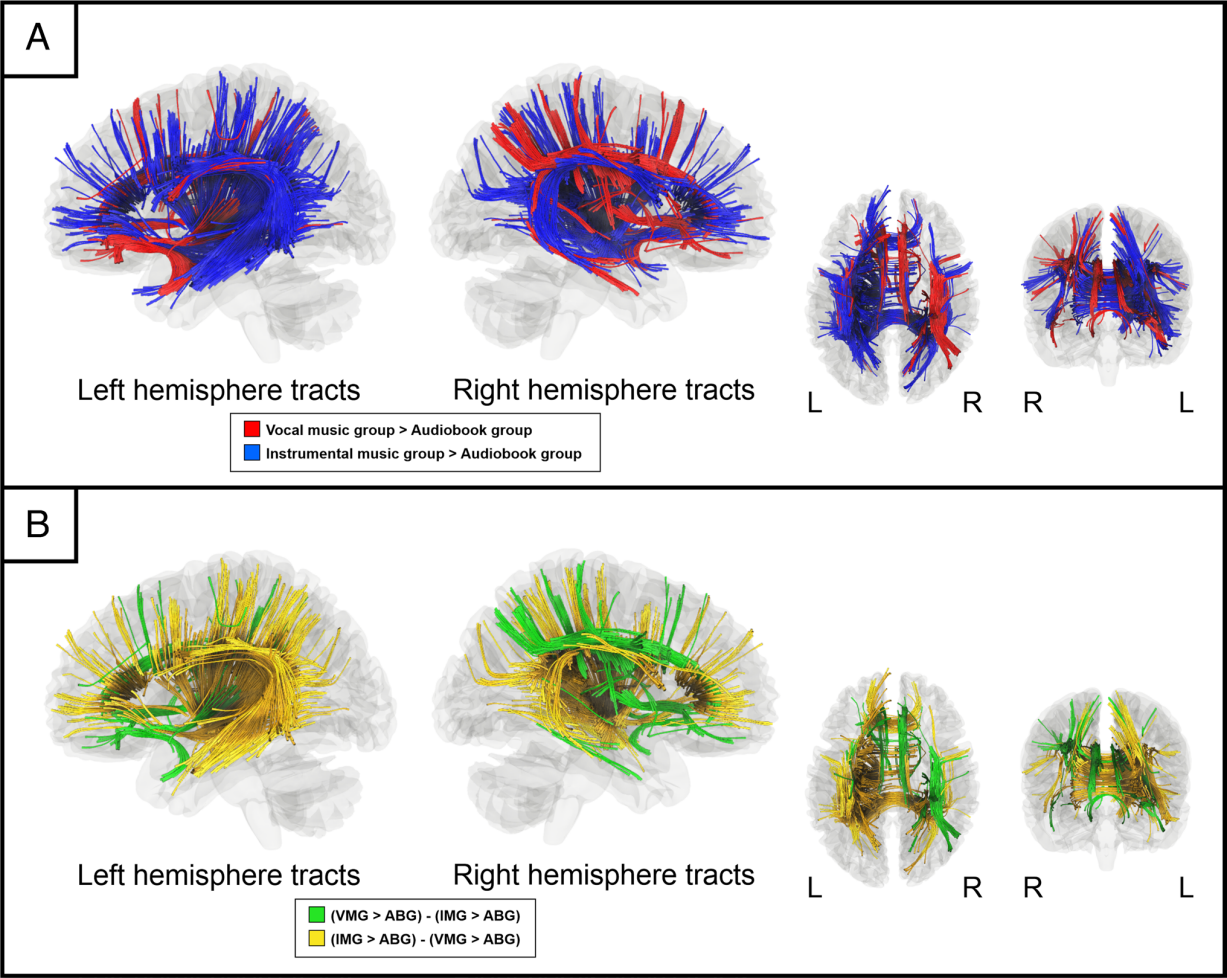
Comparison of significant connectometry findings. (**A**) Positively associated white matter tracts for VMG > ABG (red) and IMG > ABG (blue). (**B**) Positively associated white matter tracts for VMG only [(VMG > ABG) – (IMG > ABG)] (green) and IMG only [(IMG > ABG) – (VMG > ABG)] (yellow). ABG = Audiobook group, IMG = Instrumental music group, L = left, QA = Quantitative anisotropy, R = right, VMG = Vocal music group

The connectometry analyses comparing the longitudinal QA changes between the intervention groups revealed that the VMG showed greater QA increase (3 months > Acute) compared to the ABG in the left ventral (uncinate fasciculus, inferior fronto-occipital fasciculus, extreme capsule) and dorsal (arcuate fasciculus, frontal aslant tract) pathways, in the left cingulum, thalamic radiation and corticostriatal tracts as well as in the right ventral (inferior longitudinal fasciculus) and dorsal (arcuate fasciculus, superior longitudinal fasciculus) pathways, in the right cingulum, thalamic radiation and corticostriatal tracts and the corpus callosum (FDR = 0.02; Fig. 1A, Fig. 2A).

The IMG also showed greater QA increased compared to the ABG in the left ventral (uncinate fasciculus, inferior fronto-occipital fasciculus, inferior longitudinal fasciculus) and dorsal (arcuate fasciculus) pathways, in the left thalamic radiation and corticospinal and -pontine tracts as well as in the right dorsal (arcuate fasciculus, superior longitudinal fasciculus) pathway and the corpus callosum compared to the ABG (FDR = 0.0004; Fig. 1B, Fig. 2A).

Compared to the VMG or IMG, the ABG did not show significant increases in QA over time. Moreover, the comparisons between the VMG and IMG revealed no significant differences.

When the two significant connectometry findings were further compared, VMG only [(VMG > ABG) – (IMG > ABG)] was associated with increased QA in the left anterior ventral and right dorsal and ventral pathways as well as in the bilateral cingulum (see Fig. 2B) whereas IMG only [(IMG > ABG) – (VMG > ABG)] was associated with increased QA in the left posterior ventral and dorsal pathways as well as corpus callosum and the right dorsal pathway (see Fig. 2B).

## Discussion

This study set out to determine the structural connectome changes induced by post-stroke music listening in the context of auditory EE. Our novel findings were that compared to listening to audiobooks, both daily listening to vocal and instrumental music after stroke enhanced structural connectivity in dorsal and ventral pathways in both hemispheres as well as in the corpus callosum. The present study extends previous results on the rehabilitative effects of music listening after stroke (Baylan et al., 2020; Särkämö et al., 2008, 2014; Sihvonen et al., 2020), including the previously reported results from the current RCT (Sihvonen, Pitkäniemi et al., 2021; Sihvonen, Ripollés et al., 2021), and provides new information about the extent of structural connectome changes after music listening intervention. The results conform with evidence derived from animal studies (van Praag et al., 2000) showing that exposure to EE can elicit neuroanatomical changes in the white matter also in human stroke patients.

In clinical practice, successful post-stroke rehabilitation and functional restoration are grounded on neuroplasticity of the injured brain (Carmichael, 2006; Cramer, 2008, 2018; Cramer et al., 2011; Krakauer et al., 2012; Nudo, 2013). The neuroplasticity changes are driven by activity-dependent mechanisms (Cramer et al., 2011; Murphy & Corbett, 2009) that, according to animal studies, can be enhanced via EE: In animal models for stroke, EE has led to, for example, decreased lesion volume (Buchhold et al., 2007; Zhang et al., 2017) and white matter damage (Hase et al., 2018) as well as to increased synaptogenesis (Hirata et al., 2011) and axonal remodelling (Li et al., 2015). Moreover, the neural benefits of EE have been associated with improved cognitive and motor recovery (Buchhold et al., 2007; Farrell et al., 2001). Despite promising evidence derived from animal research, there has been limited translation of this intervention into patient studies (Livingston-Thomas et al., 2016) and its neural effects of have remained unexplored in human stroke patients. Previous studies have shown that a communal and individual EE increases activity in stroke patients (Janssen et al., 2014; Rosbergen et al., 2017, 2019; but see Janssen et al., 2021), but evidence on its effect on functional and cognitive recovery has remained inconsistent (Janssen et al., 2021; Khan et al., 2016). To our best knowledge, this is the first study to evaluate the effects of auditory EE (i.e., music listening), on whole-brain white matter connectivity in stroke patients. Based on the results, both vocal and instrumental music listening increase structural white matter connectivity in the post-stroke brain, providing a fertile ground for recovery (Murphy & Corbett, 2009; Särkämö & Soto, 2012).

To maximize the utilization of the activity-dependent mechanisms of brain plasticity, the rehabilitative stimulation, also in EE, needs to take place in the acute and early subacute post-stroke stages with elevated brain plasticity as well as to be intensive enough and stimulate the impaired neural network (Bernhardt et al., 2017; Foley et al., 2012; Krakauer et al., 2012; Murphy & Corbett, 2009). Failing to meet these demands could partly explain the lack of consistent clinical findings in previous EE studies in stroke patients. In the present study, patients listened to music for at least one hour per day from the early subacute stage to 3-month stage, within the time window of heightened neuroplasticity. Compared to audiobooks, both vocal and instrumental music listening induced widespread connectome changes. This likely owes to music’s capacity to induce widespread activations in the brain (Alluri et al., 2012; Koelsch, 2014; Samson et al., 2011; Schmithorst, 2005; Zatorre & Salimpoor, 2013), even in the current sample of stroke patients at the baseline before intervention (i.e., in the early subacute stage) (Sihvonen et al., 2017b). Moreover, music is a complex stimulation for the brain, involving, for example, acoustic analysis, auditory memory, auditory scene analysis, processing of interval relations, of musical syntax and semantics, and activation of motor representations of actions (Koelsch, 2011; Koelsch & Siebel, 2005). This has been suggested to stimulate multiple functional networks in the brain (Särkämö & Sihvonen, 2018), offering an avenue to tap into the activity-dependent neuroplasticity mechanisms of recovery.

In fact, the connectome changes induced by instrumental music seem more extensive compared to those induced by listening to vocal music. However, the locus of neuroplasticity changes is also important and depends on the stimulated networks. The activation patterns are more widespread when listening to music containing singing than mere instrumental music in both healthy subjects (Alluri et al., 2013; Brattico et al., 2011). Similar activation patterns were observed in the present sample of stroke patients at the early subacute stage (Sihvonen et al., 2017b). Moreover, listening to vocal music combines processing of linguistic and musical information into a unified representation, providing enhanced modulatory effects compared to processing of mere musical information (i.e., instrumental music). This improved modulation has been shown to translate into improved cognitive and neural recovery after stroke (Särkämö et al., 2008, 2014; Sihvonen et al., 2020; Sihvonen, Pitkäniemi et al., 2021; Sihvonen, Ripollés et al., 2021). In the present study, vocal music induced more neuroplasticity changes in the left anterior ventral and right dorsal and ventral pathways as well as in the bilateral cingulum as compared to instrumental music. This provides a plausible explanation for the emotional and cognitive benefits of daily music listening music post-stroke (Baylan et al., 2020; Särkämö et al., 2008), and more specifically for the cognitive improvements observed after vocal music listening in the present study (Sihvonen et al., 2020).

Studies on stroke patients have revealed that patients remain mostly inactive, alone and unstimulated during the critical acute and early subacute stage (Bernhardt et al., 2004; De Wit et al., 2005) and receive therapeutic interventions less than recommended (Foley et al., 2012), thus failing to exploit the critical periods for neuroplasticity (Cramer et al., 2011). Increased levels of stimulation for early subacute stroke patients could potentially lead to improved outcomes. Music listening could be implemented, regardless of the severity of patient’s neurological impairment, with reasonable intensities, even in inpatient wards without constant input from the staff (i.e., therapists).

The present study provides proof of concept that post-stroke music listening in the context of auditory EE could induce wide-spread changes in the structural connectome but has some potential limitations. The sample size remains modest, and the present results cannot be generalized without reservations and future larger-scale studies. As studies on the effects of post-stroke EE on the brain structure are lacking, the extent of the connectivity changes induced by music listening cannot be compared to those induced by, for example, communal enrichment. Future rehabilitation studies on auditory enrichment with more detailed estimations of the microstructural complexity of neurites using, for example, neurite orientation dispersion and density imaging (NODDI), could provide more specific information of the specific mechanisms of recovery (Zhang et al., 2012). Moreover, as the neuroplasticity changes are based on myriads of molecular changes, including those related to stress, studies on biochemical mediators of post-stroke stress are needed to further understand how music improves recovery post-stroke.

## Conclusions

In conclusion, the present connectometry results suggest that the positive effects of music listening on post-stroke recovery are underpinned by wide-spread structural reorganization, and further substantiates the conclusion that listening to music provides a fertile ground for functional restoration after stroke.

## Data Availability

Not applicable.
